# Functional Interactions between Sensory and Memory Networks for Adaptive Behavior

**DOI:** 10.1093/cercor/bhab160

**Published:** 2021-06-29

**Authors:** Vasilis M Karlaftis, Joseph Giorgio, Elisa Zamboni, Polytimi Frangou, Reuben Rideaux, Joseph J Ziminski, Zoe Kourtzi

**Affiliations:** Department of Psychology, University of Cambridge, Cambridge, UK; Department of Psychology, University of Cambridge, Cambridge, UK; Department of Psychology, University of Cambridge, Cambridge, UK; Department of Psychology, University of Cambridge, Cambridge, UK; Department of Psychology, University of Cambridge, Cambridge, UK; Department of Psychology, University of Cambridge, Cambridge, UK; Department of Psychology, University of Cambridge, Cambridge, UK

**Keywords:** adaptation, fMRI, functional connectivity, GABAergic inhibition, repetition suppression

## Abstract

The brain’s capacity to adapt to sensory inputs is key for processing sensory information efficiently and interacting in new environments. Following repeated exposure to the same sensory input, brain activity in sensory areas is known to decrease as inputs become familiar, a process known as adaptation. Yet, the brain-wide mechanisms that mediate adaptive processing remain largely unknown. Here, we combine multimodal brain imaging (functional magnetic resonance imaging [fMRI], magnetic resonance spectroscopy) with behavioral measures of orientation-specific adaptation (i.e., tilt aftereffect) to investigate the functional and neurochemical mechanisms that support adaptive processing. Our results reveal two functional brain networks: 1) a sensory-adaptation network including occipital and dorsolateral prefrontal cortex regions that show decreased fMRI responses for repeated stimuli and 2) a perceptual-memory network including regions in the parietal memory network (PMN) and dorsomedial prefrontal cortex that relate to perceptual bias (i.e., tilt aftereffect). We demonstrate that adaptation relates to increased occipito-parietal connectivity, while decreased connectivity between sensory-adaptation and perceptual-memory networks relates to GABAergic inhibition in the PMN. Thus, our findings provide evidence that suppressive interactions between sensory-adaptation (i.e., occipito-parietal) and perceptual-memory (i.e., PMN) networks support adaptive processing and behavior, proposing a key role of memory systems in efficient sensory processing.

## Introduction

Efficient processing of the diverse information sources that reach our senses relies on our ability to adapt to repeated exposure to the same sensory inputs. Perceptual aftereffects demonstrate how sensory adaptation alters human behavior. For example, consider the tilt aftereffect: Following prolonged presentation of a tilted bar (the adaptor), observers perceive a vertical bar as tilted away from the orientation of the adaptor (for review Clifford 2002). Sensory adaptation has been shown to relate to reduction in 1) neuronal responses to the features of the adaptor, as measured by electrophysiology (for review Kohn 2007) and 2) blood oxygen level-dependent (BOLD) responses to low-level features (e.g., contrast, orientation, motion; for review Larsson et al. 2016) in visual cortex due to stimulus repetition, as measured by functional brain imaging. Further, repetition suppression (i.e., decreased BOLD for repeated stimuli) has been reported in higher visual areas for repeated presentation of more complex visual stimuli (e.g., faces, objects) (Grill-Spector et al. 2006; Krekelberg et al. 2006). In contrast, repetition enhancement (i.e., increased BOLD for repeated compared to novel stimuli) has been reported in parietal, temporal, and frontal regions known to be involved in memory rather than sensory processes (for review Segaert et al. 2013). These findings showing repetition suppression in sensory areas versus repetition enhancement in memory-related regions in response to repeated stimuli have not yet been reconciled, leaving a gap in our understanding of the brain-wide mechanisms that support adaptive processing.

Here, we interrogate brain-wide functional networks involved in visual adaptation by combining functional brain imaging with a classic behavioral paradigm for measuring the tilt aftereffect. We hypothesize that both sensory and memory-related regions are involved in visual adaptation and that functional interactions between these regions mediate adaptive processing. Using functional connectivity analyses of task-based fMRI signals, we test for brain networks that are involved in 1) visual adaptation (i.e., processing of repeatedly presented stimuli) and 2) perceptual adaptation, as measured by the tilt aftereffect.

Further, previous pharmacological studies in humans (Bunzeck and Thiel 2016) and monkeys (Kuravi and Vogels 2018) have implicated GABAergic inhibition in repetition suppression. Recent advances in magnetic resonance spectroscopy (MRS) allow us to noninvasively measure GABA, the primary inhibitory neurotransmitter in the brain. Previous MRS studies have interrogated the role of GABAergic inhibition in visual processing showing that visual cortex GABA relates to behavior in visual discrimination tasks (e.g., orientation discrimination (Edden et al. 2009; Song et al. 2017). Here, we use MRS to test the role of GABAergic inhibition in sensory and memory networks involved in adaptive processing.

We demonstrate that fMRI responses in visual areas (V1, V2, V3) and dorsolateral prefrontal cortex (dlPFC) are lower for repeated than novel stimuli, consistent with fMRI repetition suppression. In contrast, fMRI responses in the parietal memory network (PMN: angular gyrus, precuneus) and dorsomedial prefrontal cortex (dmPFC) relate to perceptual adaptation, as measured by the tilt aftereffect. Further, functional connectivity analysis reveals a sensory-adaptation network (including V1, intraparietal sulcus: IPS) that is involved in the processing of repeated stimuli, while a perceptual-memory network (including PMN) that is involved in perceptual adaptation. These networks exhibit increased within-network connectivity, while decreased between-network connectivity for visual adaptation. Further, our MRS results shed light on the role of GABAergic inhibition in the interactions between sensory-adaptation and perceptual-memory networks, showing that lower GABA+ in the PMN relates to 1) decreased fMRI activity in parietal cortex (i.e., IPS) and 2) decreased connectivity between the two networks for repeated stimuli. Thus, our findings suggest that suppressive interactions between sensory-adaptation and perceptual-memory networks support adaptive processing and behavior.

## Materials and Methods

### Participants

Thirty healthy participants (16 female, 14 male) participated in the study. All participants took part in one behavioral session and two MRI scans (fMRI, MRS). Due to technical issues, behavioral data were not collected for two participants. MRI data from four participants were excluded from further analysis due to head movement-related artifacts, resulting in twenty-six participants (mean age: 25 years and SD: 4 years). All participants had normal or corrected-to-normal vision, did not receive any prescription medication, were naïve to the aim of the study, gave written informed consent, and received payment for their participation (£7/h for behavioral and £10/h for imaging sessions). The study was approved by the University of Cambridge Ethics Committee [PRE.2017.57].

### Experimental Design

All participants took part in one behavioral session and two MRI scans (fMRI, MRS). The behavioral session was conducted prior to the MRI sessions (time between sessions, mean: 6.4 days, SD: 5.9 days) and MRI scans were conducted in a counterbalanced order (time between scans, mean: 7.8 days, SD: 7.2 days).

### MRI Acquisition

We collected MRI data on a 3T Siemens PRISMA scanner (Wolfson Brain Imaging Unit, Cambridge) using a 32-channel head coil. T1-weighted structural data (MPRAGE; repetition time [TR] = 2 s; echo time [TE] = 2.98 ms; number of slices = 176; voxel size = 1 mm isotropic) and echo-planar imaging (EPI) data (gradient echo-pulse sequences) were acquired during task (TR = 0.727 s; TE = 34.6 ms; number of slices = 72; voxel size = 2 mm isotropic; Multi-Band factor = 8; flip angle = 48°; number of volumes = 405; duration = 4m54s; whole brain coverage).

We collected MRS data on a 3T Siemens PRISMA scanner (Cognition and Brain Sciences Unit, Cambridge) using a 32-channel head coil and a MEGA-PRESS sequence (Mescher et al. 1998): TE = 68 ms, TR = 3000 ms; 256 transients of 2048 data points were acquired in 13-min experiment time; a 14.28-ms Gaussian editing pulse was applied at 1.9 (ON) and 7.5 (OFF) ppm; water unsuppressed 16 transients. Water suppression was achieved using variable power with optimized relaxation delays and outer volume suppression. We conducted automated shimming followed by manual shimming. We acquired spectra from two MRS voxels (25 × 25 × 25 mm^3^): in early visual cortex (EV) and in PMN (Supplementary Fig. S1A). We manually positioned the MRS voxels using anatomical landmarks on each participant’s T1 scan, ensuring that voxel placement was consistent across participants. The EV voxel was placed medially in the occipital lobe with the lower face aligned with the cerebellar tentorium and as posterior as possible towards the occipital pole given the voxel dimensions. The PCC voxel was placed in the medial parietal lobe and rotated in the sagittal plane to align with a line connecting the genu and splenium of the corpus callosum. The center of gravity for the EV voxel was: *x* = 0.5 ± 1.7 mm, *y* = −80.4 ± 2.6 mm, *z* = 8.0 ± 3.6 mm in Montreal Neurological Institute (MNI) space, and for the PMN voxel was: *x* = −0.1 ± 1.2 mm, *y* = −51.9 ± 2.9 mm, *z* = 35.8 ± 1.7 mm in MNI space. The order of the voxels was counterbalanced across participants. During the MRS acquisition, participants fixated on a cross in the middle of the screen to encourage similar levels of alertness across participants.

### Stimuli

Stimuli comprised sinewave gratings (1 cycle/degree) of varying orientations, presented within an annulus aperture (inner radius, 0.21°; outer radius, 6°). The outer edge of the aperture was smoothed according to a sinusoidal function (SD, 0.6°). Stimuli were presented centrally, on a mid-gray background. Experiments were controlled using MATLAB and the Psychophysics toolbox 3.0 (Brainard 1997; Pelli 1997). For the fMRI session, stimuli were presented using a projector and a mirror setup (1920 × 1080 resolution, 60 Hz frame rate) at a viewing distance of 72 cm. For the behavioral session, stimuli were presented on a 21-inch CRT monitor (ViewSonic P225f, 1280 × 800 resolution, 85 Hz frame rate) at a viewing distance of 50 cm. For both the fMRI and behavioral tests, the viewing distance was adjusted so that angular stimulus size was constant during behavioral and scanning sessions.

#### Behavioral Session

We used a tilt aftereffect paradigm to test for perceptual adaptation. Participants were tested on two conditions: 1) Adaptation: sinewave gratings were presented repeatedly at the same orientation (−15° or +15° from vertical, each orientation was tested for 7 blocks in random order); 2) non-adaptation: the orientation of the gratings varied based on a uniform distribution ranging from −85° to −5° and +5° to +85°, excluding vertical (i.e., 0°). Participants completed a minimum of 2 and maximum of 5 runs, each comprising 14 adaptation and 14 non-adaptation blocks. Adaptation and non-adaptation blocks were presented in alternating order.

For each block, participants were exposed to 21 sample stimuli that were presented sequentially and were followed by a test stimulus (orientation randomly selected between ±5.3° from vertical). Each sample stimulus was displayed for 1300 ms with an inter-stimulus interval of 154 ms. Each test stimulus was presented for 200 ms. Participants were asked to judge whether the test stimulus was oriented clockwise or anticlockwise with respect to vertical. During the presentation of the sample stimuli, participants performed the same RSVP task as during scanning.

Further, following previous work implicating memory-related networks in repetition enhancement (for review Segaert et al. 2013), we tested participants in a visual short-term memory task (Luck and Vogel 1997). Participants were asked to memorize the color of dots in the first presentation and judge whether the color of a target dot changed in the second presentation. The colored dots (12-pixel radius) were displayed in random locations on a gray background for 500 ms, followed by a 1000 ms delay. The second presentation remained on the screen until participants responded. The number of presented dots was controlled by a 2-down-1-up staircase procedure, starting with 2 colored dots and finishing at 10 staircase reversals (converging at 70.7% performance). Participants’ memory score was measured as the average of the last 6 reversals.

#### fMRI Scan

**Figure 1:**
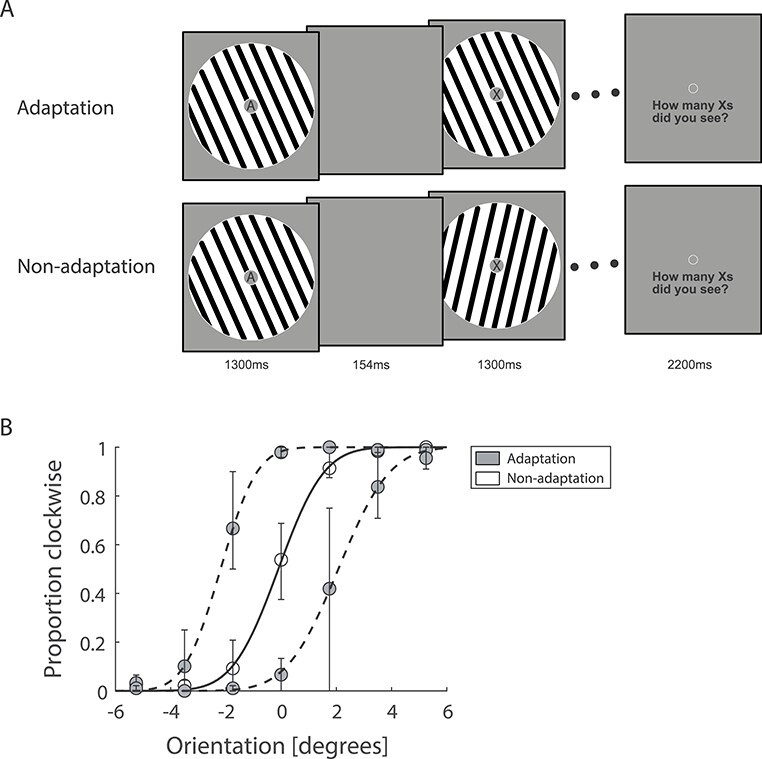
fMRI design and behavioral results. (*A*) fMRI experiment comprised 3 adaptation blocks (21 sinewave gratings presented at the same orientation) and 3 non-adaptation blocks (21 sinewave gratings presented at different orientations). During stimulus presentation, participants were instructed to perform an RSVP task; that is, count the number of times a target letter (e.g., *X*) was displayed in the stream of distractors and report it at the end of each stimulus block. (*B*) Perceptual adaptation was measured using a tilt aftereffect paradigm. We presented participants with the same stimuli (oriented gratings) as in the fMRI experiment in adaptation versus non-adaptation blocks. Participants were asked to perform the same RSVP task as in the fMRI experiment. Each block was followed by a test stimulus (a single grating with orientation close to vertical). Participants were then asked to judge whether the test stimulus was tilted clockwise or anticlockwise relative to vertical. We fitted psychometric functions to the participant responses. Solid line indicates the fit of the data in the non-adaptation condition, and dashed lines indicate the fit of the data in the adaptation conditions. Error bars indicate the first and third quartiles of the data distribution across participants (*N* = 24). Participant responses showed a perceptual bias, that is, a shift in the perceived orientation of the test stimulus for the adaptation compared to the non-adaptation condition.

The fMRI scan comprised 8 runs. Each run started with a 13.8-s fixation block, followed by 6 stimulus blocks, 3 blocks per condition (adaptation, non-adaptation) (Fig. 1*A*). The order of the blocks was counterbalanced within and across runs. Each block comprised 21 stimuli followed by 2.2 s for response and 13.8 s of fixation before the start of the next block. Each run ended with a 13.8 s fixation block. The adaptation condition comprised 21 gratings presented at the same orientation. The same orientation was presented across adaptation blocks per participant and was selected randomly from a uniform distribution, ranging from −85° to −5°, and +5° to +85°, excluding vertical (i.e., 0°). The non-adaptation condition comprised 21 gratings presented at different orientations drawn randomly from a uniform distribution within ±85° from vertical. Each stimulus was displayed for 1300 ms with a jittered inter-stimulus interval (0–154 ms) for both the adaptation and non-adaptation conditions to ensure similar stimulus presentation parameters (e.g., stimulus transients) between conditions. Since expectation and attention can impede the detection of repetition effects or alter their direction (Segaert et al. 2013; Larsson et al. 2016), we used a secondary attentional task at fixation to divert participant’s attention away from the stimulus. In particular, participants engaged in a Rapid Serial Visual Presentation (RSVP) task. A stream of letters was presented in rapid serial order (presentation frequency: 250 ms, asynchronous with the timings of grating presentation) within the inner aperture of the sinewave annulus at the center of the screen (0.42° of visual angle). Participants were asked to fixate at the inner aperture and report the number of targets (1–4 per block) by a key press when prompted at the end of each block. No feedback was provided to the participants.

### Data Analysis

#### Behavioral Data Analysis

We measured perceptual adaptation in the context of a tilt aftereffect paradigm by computing psychometric functions based on the participants’ responses to the test stimulus for both conditions (adaptation, non-adaptation). We plotted the proportion of clockwise responses as a function of test orientation and used psignifit (MATLAB, Toolbox for Bayesian psychometric function estimation) to fit a sigmoid psychometric function. We computed a perceptual adaptation index as the difference between the estimated mean parameter of the fitting functions for the adapted and non-adapted conditions (Rokem et al. 2011). For the RSVP task, we computed the percentage of accurately detected targets across runs for each condition (adaptation, non-adaptation).

### MRI Data Analysis

#### Preprocessing

We preprocessed the task-based fMRI data in SPM12.3 (v6906; www.fil.ion.ucl.ac.uk/spm/software/spm12/) following the Human Connectome Project (HCP) pipeline for multiband data (Smith et al. 2013). In particular, we first coregistered (nonlinear) the T1w structural images (after brain extraction) to MNI space to ensure that all participant data were in the same stereotactic space for statistical analysis. We then 1) corrected the EPI data for any spatial misalignments between EPI volumes due to head movement (i.e., aligned each run to its single band reference image), 2) coregistered all 8 EPI runs to the first run (rigid body) to correct any spatial misalignments between runs, 3) coregistered the first EPI run to the structural image (rigid body), and 4) normalized them to MNI space for subsequent statistical analyses (applying the deformation field of the structural images). Data were only resliced after MNI normalization to minimize the number of interpolation steps. Following MNI normalization, data were skull-stripped, spatially smoothed with a 4-mm Gaussian kernel to improve the signal-to-noise ratio and the alignment between participant data (two times the voxel size; Chen and Calhoun 2018), and had linear drifts removed (linear detrending due to scanner noise). Slice-timing correction was not applied, following previous work on fast TR (subsecond) acquisition protocols (Smith et al. 2013).

Next, we applied spatial group independent component analysis (ICA) using the Group ICA fMRI Toolbox (GIFT v3.0b) (http://mialab.mrn.org/software/gift/) to identify and remove components of noise. Principal component analysis was applied for dimensionality reduction, first at the subject level, then at the group level. The Minimum Description Length criteria (Rissanen 1978) were used to estimate the dimensionality and determine the number of independent components, resulting in 34 independent components. The ICA estimation (Infomax) was run 20 times and the component stability was estimated using ICASSO (Himberg et al. 2004). Following recent work on back-reconstruction methods for ICA denoising at the group level (Du et al. 2016), we used Group Information-Guided ICA (GIG-ICA) back-reconstruction to reconstruct subject-specific components from the group components. We visually inspected the results and identified noise components according to published procedures (Griffanti et al. 2017). Using consensus voting among 3 experts (V.M.K., J.G., P.F.), we labeled 17 of the 34 components as noise that captured signal from veins, arteries, cerebrospinal fluid (CSF) pulsation, susceptibility, and multiband artifacts.

To clean the fMRI signals from motion artifacts and the noise components, we followed a soft cleanup ICA denoise approach (Griffanti et al. 2014). That is, we first regressed out the motion parameters (translation, rotation, and their squares and derivatives; Friston et al. 1996) from each voxel and ICA component time course. Second, we estimated the contribution of each ICA component to each voxel’s time course (multiple regression). Finally, we subtracted the unique contribution of the noise components from each voxel’s time course to avoid removing any shared signal between neuronal and noise components. The residual time course from the last step was used for all subsequent BOLD and connectivity analyses.

### BOLD Analysis

We performed a first-level analysis, modeling two task conditions (adaptation, non-adaptation) across runs per participant. We selected the hemodynamic model with derivatives as basis functions to account for individual differences in latency and width of the hemodynamic response (Friston et al. 1998). Within the GLM, the data were high-pass filtered at 0.01 Hz and treated for serial correlations using the FAST autoregressive model, as it has been shown to perform more accurate autocorrelation modeling for fast TR acquisitions (Corbin et al. 2018; Olszowy et al. 2019). We then computed boosted betas per participant and condition (Pernet 2014) and entered them into a second-level analysis model. We tested for 1) voxel-wise BOLD differences between conditions, 2) BOLD differences between conditions that correlate with behavior (i.e., group GLM with perceptual adaptation index as regressor), and 3) BOLD differences between conditions that correlate with local GABAergic inhibition as measured by MRS (i.e., group GLM with EV or PMN GABA+ as regressor). All voxel-wise statistical analyses were thresholded at *P* < 0.005 uncorrected (i.e., cluster-extent) and cluster-corrected at *P* = 0.05 family-wise error rate (FWER), unless otherwise stated. Significant clusters were labeled using the probabilistic map of visual topography (Wang et al. 2015) or the automated anatomical labeling (AAL) and HCP atlases (Tzourio-Mazoyer et al. 2002; Glasser et al. 2016) for clusters beyond the visual cortex.

### Functional Connectivity Analysis

We deconvolved the voxel time course using finite impulse response functions following recent work (Cole et al. 2019) by fitting 63 regressors per condition capturing the stimulus block (30.5 s), response period (2.2 s), and fixation block (13.8 s). This method allowed us to accurately model and remove the cross-block mean response for each task condition (adaptation, non-adaptation), accounting for potential task-timing confounds that have been shown to inflate the strength of the computed task-based functional connectivity (Cole et al. 2019). Within the GLM, the data were treated for serial correlations using the FAST autoregressive model (Corbin et al. 2018; Olszowy et al. 2019). For each region of interest (ROI), we computed the first eigenvariate across all voxels within the region to derive a single representative time course per ROI.

First, we performed seed-based connectivity analysis to investigate the connectivity of each ROI to the rest of the brain. That is, for each block, we extracted the time course per ROI, applied high-pass filtering at 0.0328 Hz, and correlated the ROI time course with every voxel in the brain (Pearson correlation). The frequency of the filter was selected based on previous work (Leonardi and Van De Ville 2015), suggesting that the cutoff should be equal to 1/window_length, where window_length = 30.5 s for each task block. We subsequently averaged the connectivity maps across blocks and runs (after Fisher-*z* transformation) to derive a single connectivity map per participant per condition. We then tested for 1) group differences in seed-based connectivity between conditions and 2) connectivity differences between conditions that correlate with local GABAergic inhibition (i.e., EV or PMN GABA+) as measured by MRS. All voxel-wise statistical analyses were thresholded at *P* < 0.005 uncorrected (i.e., cluster-extent) and cluster-corrected at *P* = 0.05 FWER, unless otherwise stated. Significant clusters were labeled using the probabilistic map of visual topography (Wang et al. 2015) or the AAL and HCP atlases (Tzourio-Mazoyer et al. 2002; Glasser et al. 2016) for clusters beyond the visual cortex.

### MRS Analysis

We preprocessed the MRS data using MRspa v1.5c (www.cmrr.umn.edu/downloads/mrspa/). We applied Eddy current, frequency, and phase correction before subtracting the average ON and OFF spectra, resulting in edited spectra. We used LC-Model (Provencher 2001) to quantify metabolite concentrations by fitting model spectra of γ-aminobutyric acid (GABA), Glutamate (Glu), Glutamine (Gln), and N-acetylaspartate (NAA) to the edited spectra (Supplementary Fig. S1B). We refer to GABA concentration as GABA+, as MRS measurements of GABA with MEGA-PRESS include co-edited macromolecules (Mullins et al. 2014). We referenced GABA+ and Glu concentrations to the concentration of NAA (GABA+/NAA) and used GABA+ referenced to water (GABA+/water) to ensure that our results were not driven by the chosen reference (Lunghi et al. 2015).

Further, we conducted whole brain tissue-type segmentation of the T1-weighted structural scan and calculated the percentage of gray matter, white matter, and CSF in each MRS voxel. To ensure that correlations with GABA+ were not driven by variability in tissue composition within the MRS voxel across participants, we conducted two control analyses (Supplementary Table S2): 1) regressed out the CSF percentage from the GABA+ concentrations and 2) applied α-correction on the GABA+/water values to account for the difference in GABA+ between gray and white matter (Porges et al. 2017).

All spectra had linewidth below 10 Hz and GABA+ Cramer-Rao lower bound (CRLB) values smaller than 10%. Data for two participants were excluded due to lipid contamination, as detected by visual inspection by two independent reviewers (P.F., J.J.Z.). Signal-to-noise ratio (SNR) was calculated as the amplitude of the NAA peak in the difference spectrum divided by twice the root mean square of the residual signal (Provencher 2001). To control for potential differences in data quality across participants, we performed control analyses that accounted for variability in absolute CRLB (Kreis 2016), linewidth, and SNR across participants (Supplementary Tables S1 and S2).

## Results

### Behavioral Adaptation

We measured perceptual adaptation using an established tilt aftereffect paradigm (Larsson et al. 2006). We tested for perceptual bias due to adaptation, as indicated by a shift in the perceived orientation of a vertical test stimulus following adaptation (i.e., prolonged exposure to a tilted grating). We observed a significantly higher shift (*t*(23) = 16.61, *P* < 0.0001; Fig. 1*B*) in the perceived orientation of the test stimulus after adaptation to a leftward (mean = −2.21°) or rightward orientated grating (mean = 2.17°) compared to non-adaptation (i.e., exposure to gratings that varied in their orientation within a range of ±85° from vertical, mean = −0.07°). This perceptual bias due to adaptation is consistent with higher perceptual sensitivity to novel than repeated stimuli (Clifford 2002; Webster 2011).

### fMRI Adaptation and Its Relationship to Perceptual Adaptation

**Figure 2:**
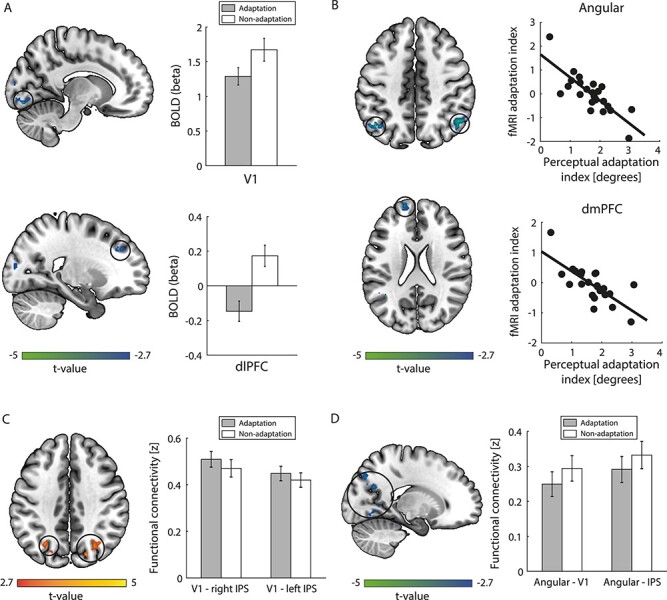
BOLD and functional connectivity analyses. Brain maps show significant clusters (FWER cluster-corrected at *P* = 0.05, highlighted by circles) and are displayed in neurological convention (left is left). (*A*) BOLD GLM analysis. On the left, clusters in V1 (slice: *x* = 12) and dlPFC (slice: *x* = 22) showing lower BOLD for adaptation than non-adaptation (Table S3). On the right, BOLD response (beta) is shown per condition (adaptation, non-adaptation) for these clusters. (*B*) BOLD GLM analysis with behavior as covariate. On the left, clusters in angular (slice: *z* = 44) and dmPFC (slice: *z* = 22) showing a negative correlation of fMRI adaptation index (BOLD for adaptation minus non-adaptation) with behavior (Table S4). On the right, scatterplot of fMRI adaptation index with perceptual adaptation index is shown for these clusters. All correlations remained significant after controlling for the time interval between behavioral and MRI sessions (*P* < 0.001). (*C*) Seed-based functional connectivity from V1. On the left, clusters (slice: *z* = 38) showing higher connectivity for adaptation than non-adaptation (Table S5). On the right, barplots of V1 connectivity (Fisher’s *z*) are shown per condition for V1—right IPS and V1—left IPS connectivity. (*D*) Seed-based functional connectivity from angular gyrus. On the left, clusters (slice: *x* = −18) showing lower connectivity for adaptation than non-adaptation (Table S6). On the right, barplots of angular gyrus (Fisher’s *z*) connectivity are shown per condition for Angular—V1 and Angular—IPS connectivity. Error bars indicate standard error of the mean across participants.

We performed a whole-brain GLM analysis to test for differences in fMRI responses (i.e., BOLD) between the adaptation and non-adaptation conditions. This analysis showed significantly decreased fMRI responses in visual (V1, V2, V3) and frontal (middle frontal gyrus: MFG; dorsal area 9/46 corresponding to mid-dlPFC) regions for adaptation compared to non-adaptation (Fig. 2*A*; Supplementary Table S3). This fMRI adaptation is consistent with previous studies showing decreased BOLD in visual cortex for repeated stimulus presentation (Grill-Spector et al. 2006; Larsson et al. 2016). Further, previous studies have shown that dlPFC is involved in the processing of familiar stimuli (Henson et al. 1999; Kim 2011), the abstract representation of events that are expected to occur (Petrides 2005), and is functionally connected to the visual cortex (Baker et al. 2018).

To ensure that any differences in fMRI responses between conditions (i.e., adaptation vs. non-adaptation) were not due to differences in participant’s attention, participants performed an RSVP task during scanning. That is, participants were asked to detect a target from a stream of letters presented in the center of the screen. Mean performance across participants in this task did not differ significantly between conditions (*t*(25) = −0.36, *P* = 0.72; adaptation condition: mean 78.0%, SD ±16.9%; non-adaptation condition: mean 78.7%, SD ±16.4%). Thus, it is unlikely that the fMRI adaptation we observed was due to differences in attention across tasks.

Next, we tested whether fMRI adaptation relates to perceptual adaptation. We computed a perceptual adaptation index as the difference in the perceived orientation between the adaptation and non-adaptation conditions (higher index indicates stronger perceptual bias due to adaptation). We computed an fMRI adaptation index as the difference in fMRI responses between the adaptation and non-adaptation conditions. A positive fMRI adaptation index indicates stronger fMRI responses for repeated stimuli (i.e., repetition enhancement), whereas a negative index indicates stronger fMRI responses for novel stimuli (i.e., repetition suppression). We then conducted a whole-brain GLM analysis with the perceptual adaptation index as regressor to test for fMRI differences between conditions (i.e., fMRI adaptation index) that relate to behavior. We found significant clusters in parietal (angular gyrus), temporal (middle temporal gyrus: MTG), and frontal (superior frontal gyrus: SFG; anterior area 9 corresponding to dmPFC) regions that showed a negative correlation with behavior (Fig. 2*B*; Supplementary Table S4). That is, stronger perceptual bias relates to lower fMRI responses for repeated compared to novel stimuli (i.e., repetition suppression) in these regions. We note that the angular gyrus clusters overlap with the PMN, as identified by ICA (Supplementary Fig. S2). Previous studies have shown that fMRI activity for repeated stimuli in PMN relates to memory processes (Segaert et al. 2013; Gilmore et al. 2015). Further, previous studies have shown similar fMRI adaptation effects in parieto-temporal (angular gyrus, MTG) and dmPFC regions (Kim 2011; Segaert et al. 2013) that are known to be functionally connected (Baker et al. 2018), suggesting that these regions belong to the same functional network.

This whole-brain GLM analysis with perceptual adaptation did not reveal any significant clusters in visual cortex. Further, correlating fMRI adaptation index in V1 with perceptual adaptation index did not show a significant relationship (*r*(22) = −0.18, *P* = 0.390). Previous studies have suggested a link between repetition suppression and perceptual adaptation (Engel 2005; Fang et al. 2005, 2007). Interestingly, recent laminar fMRI studies have shown that perceptual adaptation correlates with fMRI adaptation in superficial layers of early visual cortex (Ge et al. 2020; Zamboni et al. 2020). It is likely that averaging fMRI signals across cortical depths masks the layer-specific relationship between BOLD activity and perceptual adaptation index, resulting in lack of significant correlations between perceptual and fMRI adaptation in visual cortex.

To interrogate further the link between perceptual adaptation and memory, we asked whether stronger visual memory relates to stronger perceptual bias (i.e., the stronger the memory for the adaptor the further away the test stimulus is perceived to be oriented from the adaptor). Correlating participant performance in a visual short-term memory task with the perceptual adaptation index showed a significant positive correlation (*r*(15) = 0.56, *P* = 0.019), suggesting that stronger visual memory relates to stronger perceptual bias. Further, correlating visual short-term memory performance with fMRI adaptation index showed a significant negative correlation in angular gyrus (*r*(15) = −0.51, *P* = 0.035) and dmPFC clusters (*r*(15) = −0.56, *P* = 0.019), suggesting that lower fMRI responses for repeated stimuli (i.e., repetition suppression) in these regions relate to stronger visual memory.

Taken together, our results demonstrate that fMRI responses in visual and dorsolateral prefrontal regions decrease for familiar (i.e., repeated) stimuli. In contrast, fMRI responses in parieto-temporal regions and dorsomedial prefrontal regions relate to perceptual adaptation and visual memory, suggesting a role of memory processes in adaptive sensory processing.

### Functional Brain Networks Involved in Adaptive Processing

To test for functional brain networks involved in adaptive processing, we conducted whole brain functional connectivity analyses seeded from 1) the primary visual cortex (V1) cluster that showed decreased fMRI responses for adaptation than non-adaptation (1st cluster in Supplementary Table S3) and 2) the largest clusters in the angular gyrus that showed significant correlation with perceptual adaptation (union of 2nd and 3rd cluster in Supplementary Table S4). For connectivity seeded from the visual cortex, we observed two significant clusters in IPS that showed higher connectivity to V1 for adaptation compared to non-adaptation (Fig. 2*C*; Supplementary Table S5). For connectivity seeded from the angular gyrus, we observed significant clusters in the occipito-temporal and occipito-parietal cortex (including V1 and IPS) that showed lower connectivity to angular gyrus for adaptation compared to non-adaptation (Fig. 2*D*; Supplementary Table S6).

Taken together, our results suggest that repeated stimulus presentation enhances functional connectivity between visual and posterior parietal regions that are known to be involved in the expectation and detection of novel stimuli (Summerfield and De Lange 2014; de Lange et al. 2018). In contrast, repeated stimulus presentation decreases functional connectivity between occipito-parietal regions and angular gyrus that we showed to relate to perceptual adaptation.

We corroborated these results by conducting the same analysis on an independent sample of participants (*N* = 15) that participated in a 7T imaging study using the same paradigm and task (Zamboni et al. 2020). We selected two ROIs: V1 (defined by retinotopic mapping) and angular gyrus (defined anatomically to match the 2nd and 3rd clusters in Supplementary Table S4). First, we found significantly decreased fMRI responses in V1 for adaptation compared to non-adaptation (*t*(14) = −6.30, *P* < 0.0001; Supplementary Fig. S3A). Second, seed-based functional connectivity analysis (cluster-extent at *P* < 0.025 uncorrected, cluster-corrected at *P* = 0.05 FWER) from V1 showed a significant cluster in IPS with higher connectivity to V1 for adaptation compared to non-adaptation (Supplementary Fig. S3B; Supplementary Table S7). Third, seed-based functional connectivity from angular gyrus showed a significant cluster in V1 with decreased connectivity to angular gyrus for adaptation than non-adaptation (Supplementary Fig. S3C; Supplementary Table S8).

**Figure 3:**
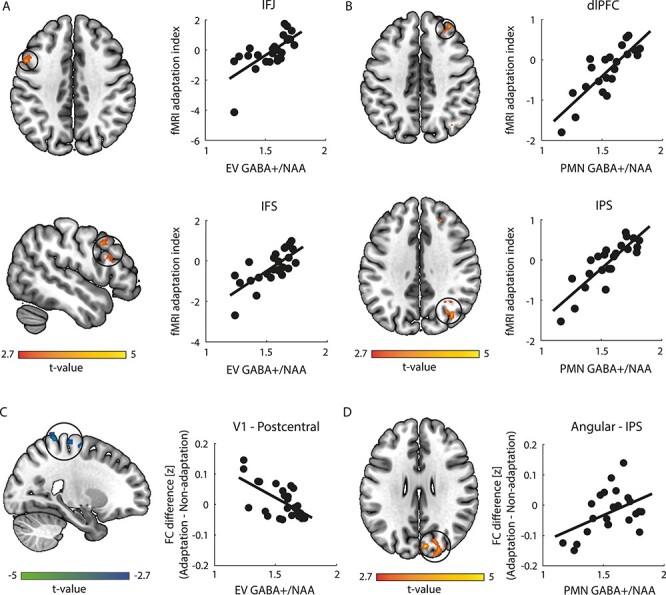
BOLD and functional connectivity analyses with GABA+ as regressor. Brain maps show significant clusters (FWER cluster-corrected at *P* = 0.05, highlighted by circles) and are displayed in neurological convention (left is left). (*A*) BOLD GLM analysis with EV GABA+ as covariate. On the left, clusters in IFJ (slice: *z* = 38) and IFS (slice: *x* = −50) showing a positive correlation of fMRI adaptation index (BOLD for adaptation minus non-adaptation) with EV GABA+ (Table S9). On the right, scatterplot of fMRI adaptation index with EV GABA+ is shown for these clusters. (*B*) BOLD GLM analysis with PMN GABA+ as covariate. On the left, clusters in dlPFC (slice: *z* = 42) and IPS (slice: *z* = 36) showing a positive correlation of fMRI adaptation index with PMN GABA+ (Table S10). On the right, scatterplot of fMRI adaptation index with PMN GABA+ is shown for these clusters. (*C*) Seed-based functional connectivity from V1 with EV GABA+ as covariate. On the left, clusters (slice: *x* = 24) showing a negative correlation of connectivity difference (Fisher’s *z*; adaptation minus non-adaptation) with EV GABA+ (Table S11). On the right, scatterplot of connectivity difference with EV GABA+ is shown for V1—postcentral connectivity. (*D*) Seed-based functional connectivity from angular gyrus with PMN GABA+ as covariate. On the left, spatial location of clusters (slice: *z* = 28) showing a positive correlation of connectivity difference with PMN GABA+ (Table S12). On the right, scatterplot of connectivity difference (Fisher’s *z*) with PMN GABA+ is shown for angular—IPS connectivity. All correlations remained significant after controlling for the time interval between the fMRI and MRS sessions (*P* < 0.007).

### Role of GABAergic Inhibition in Adaptive Processing

Previous work has shown that GABA, the primary inhibitory neurotransmitter in the brain, is involved in orientation processing (Edden et al. 2009; Song et al. 2017) and repetition suppression (Bunzeck and Thiel 2016; Kuravi and Vogels 2018). Further, recent studies have shown that GABA measured by MRS relates to fMRI activation (Donahue et al. 2010; Frangou et al. 2018) and functional connectivity (Stagg et al. 2014; Frangou et al. 2019). Here, we tested whether GABAergic inhibition in visual and PMN regions—shown by the GLM analyses to be involved in the processing of repeated stimuli—relates to changes in fMRI activity and connectivity due to adaptation.

First, we tested for regions that show differences in fMRI responses between conditions (i.e., fMRI adaptation index) related to GABAergic inhibition in EV and PMN. A whole-brain GLM analysis with EV GABA+ as regressor showed two significant clusters in frontal cortex (inferior frontal junction [IFJ], inferior frontal sulcus [IFS]) that are known to be involved in task preparation (Brass and Von Cramon 2002). Figure 3*A* (Supplementary Table S9) shows that stronger GABAergic inhibition in the EV relates to higher fMRI adaptation index (i.e., stronger fMRI responses for repeated compared to novel stimuli) in these frontal regions. This relationship remained significant when using GABA+ referenced to water, and when controlling for 1) tissue composition within the EV voxel, 2) EV Glu, and 3) MRS data quality (Supplementary Table S2). We did not observe any significant correlations between EV GABA+ and perceptual adaptation index (*r*(25) = −0.11, *P* = 0.578) nor fMRI adaptation index in the EV voxel (*r*(23) = −0.02, *P* = 0.909).

A similar whole-brain GLM analysis with PMN GABA+ as regressor showed significant clusters in parietal (IPS) and frontal (dlPFC) regions. These clusters remained significant when using GABA+ referenced to water, and when controlling for 1) tissue composition within the PMN voxel, 2) PMN Glu, and 3) MRS data quality (Supplementary Table S2). Figure 3*B* (Supplementary Table S10) shows that stronger GABAergic inhibition in the PMN network relates to higher fMRI adaptation index (i.e., stronger fMRI responses for repeated compared to novel stimuli) in IPS and dlPFC. Note that our previous analyses showed these regions to be involved in the processing of familiar (i.e., repeated) stimuli; that is, IPS showed increased connectivity to V1 for repeated stimuli (Supplementary Table S5), while dlPFC showed decreased fMRI responses for repeated stimuli (Supplementary Table S3). We did not observe any significant correlations between PMN GABA+ and perceptual adaptation index (*r*(23) = 0.02, *P* = 0.934) nor fMRI adaptation index in the PMN voxel (*r*(21) = 0.14, *P* = 0.512).

Second, we performed a whole-brain connectivity analysis seeded from V1 (as defined by the GLM analysis, Supplementary Table S3) with EV GABA+ as regressor to test for connectivity differences (i.e., adaptation minus non-adaptation) related to GABAergic inhibition in the visual cortex. We found two significant clusters that showed a negative correlation with EV GABA+ (Fig. 3*C*; Supplementary Table S11) in postcentral gyrus that is known to be involved in motor preparation (Eschen et al. 2007). This relationship remained significant when using GABA+ referenced to water, and when controlling for 1) tissue composition within the EV voxel, 2) EV Glu, and 3) MRS data quality (Supplementary Table S2).

We next performed a whole-brain connectivity analysis seeded from angular gyrus (as defined by the GLM analysis with behavior as regressor, Supplementary Table S4) with PMN GABA+ as regressor to test for connectivity differences related to GABAergic inhibition in the PMN network. We found a significant cluster in IPS that showed a positive correlation with PMN GABA+ (Fig. 3*D*; Supplementary Table S12). Note that this cluster overlaps with the IPS cluster revealed by the GLM analysis with PMN GABA+ as regressor (Supplementary Table S10) and remained significant when using GABA+ referenced to water, and when controlling for 1) tissue composition within the PMN voxel, 2) PMN Glu, and 3) MRS data quality (Supplementary Table S2). Finally, using the MRS voxels (i.e., 50% overlap across participants’ MRS voxels) as seed regions (EV, PMN) showed the same significant clusters, replicating our results (Supplementary Tables S13 and S14).

Taken together, our results demonstrate that stronger GABAergic inhibition in the EV relates to increased fMRI responses for repeated stimuli in frontal regions and decreased connectivity between visual and motor regions. In contrast, lower GABAergic inhibition in the PMN relates to decreased fMRI responses for repeated stimuli in IPS and dlPFC, and decreased connectivity between IPS and PMN regions.

## Discussion

Here, we combine multimodal brain imaging (fMRI, MRS) to investigate the functional brain network interactions involved in adaptive processing and behavior. We provide evidence for two functional networks: 1) a sensory-adaptation network that involves occipito-parietal and dlPFC regions and supports adaptive processing due to stimulus repetition and 2) a perceptual adaptation network that involves the parietal memory network (PMN) and dmPFC regions and relates to perceptual bias due to stimulus repetition. Further, we provide evidence for suppressive interactions between these networks that relate to GABAergic inhibition in the PMN, as indicated by decreased functional connectivity between these networks for familiar stimuli.

First, we show repetition suppression (i.e., decreased fMRI responses) in visual cortex and dlPFC, consistent with previous studies showing decreased fMRI and neuronal responses for orientation-specific adaptation in visual cortex (Clifford 2002; Krekelberg et al. 2006) and the role of dlPFC in processing and monitoring familiar stimuli (Henson et al. 1999; Petrides 2005; Kim 2011). Further, we show increased functional connectivity between primary visual cortex (V1) and posterior parietal cortex (i.e., IPS) for familiar stimuli. This suggests enhanced information transfer within an occipito-parietal network for visual adaptation, consistent with top-down influences to visual processing (Summerfield et al. 2008; Ewbank et al. 2011) from posterior parietal regions that are known to be involved in expectation and detection of novel stimuli (Summerfield and De Lange 2014; de Lange et al. 2018). These results are consistent with recent laminar fMRI studies providing evidence for top-down influences to visual adaptation through feedback from posterior parietal cortex to primary visual cortex (Ge et al. 2020; Zamboni et al. 2020).

Second, we show that PMN (angular gyrus, precuneus) and dmPFC regions relate to perceptual adaptation as measured by perceptual bias (i.e., tilt aftereffect). In particular, stronger perceptual bias relates to lower fMRI responses for repeated stimuli in these regions (i.e., repetition suppression). Further, we show that better performance in a visual memory task relates to stronger perceptual bias and lower fMRI responses in the PMN and dmPFC for repeated stimuli. These results are consistent with previous studies that have implicated PMN and dmPFC regions in the processing of familiar (i.e., repeated) stimuli in the context of memory retrieval tasks, suggesting that fMRI activity in these regions relates to memory processes (for review Segaert et al. 2013). Unlike these previous studies, we did not observe significant repetition enhancement in PMN regions (i.e., significantly higher BOLD for adaptation than non-adaptation), possibly due to the fact that participants in our study did not engage in a memory task during scanning. However, the significant correlations we observed between BOLD and behavior (i.e., perceptual adaptation) suggest that memory processes in PMN regions play a role in adaptive sensory processing; that is, the stronger the memory for the adaptor the stronger the perceptual bias (i.e., the further away the test stimulus is perceived to be oriented from the adaptor).

Third, we interrogated interactions between the sensory-adaptation (V1, IPS, dlPFC) and the perceptual-memory (PMN, dmPFC) networks. Our results demonstrate that the perceptual-memory network exhibits decreased functional connectivity to the sensory-adaptation network for repeated stimuli. Further, we show that this functional connectivity between networks is gated by GABAergic inhibition. Our previous work has shown that GABAergic inhibition in the occipital and parietal cortex plays a key role in learning by moderating the relationship between neuronal activity and behavior (Frangou et al. 2018, 2019). Here, we propose that GABAergic inhibition in the PMN gates network interactions for adaptive processing. In particular, we show that lower PMN GABA+ relates to stronger repetition suppression (i.e., lower fMRI responses for repeated stimuli) in IPS. This finding is in line with previous pharmacological studies showing that administration of a GABA-agonist (Lorazepam) attenuates perceptual and fMRI adaptation effects (for review Bunzeck and Thiel 2016), while administration of a GABA antagonist (Gabazine) enhances repetition suppression effects (Kuravi and Vogels 2018), suggesting that lower GABA relates to stronger repetition suppression. Further, we show that lower PMN GABA+ relates to decreased functional connectivity between PMN and IPS for repeated stimuli. Conversely, higher GABAergic inhibition in the PMN relates to stronger repetition enhancement (i.e., higher fMRI responses for repeated stimuli) in IPS and stronger functional connectivity between PMN and IPS for novel stimuli. These MRS results complement our fMRI findings and advance our understanding of the brain-wide interactions that support adaptive processing. We interpret these findings in light of previous work showing that GABAergic inhibition regulates network activity (e.g., Mann and Paulsen 2007; Bonifazi et al. 2009). That is, it is likely that GABAergic inhibition in the PMN gates connectivity between sensory-adaptation and perceptual-memory networks to support adaptive processing.

Computational approaches provide interesting perspectives for interpreting our findings. Recent modeling work (Ito et al. 2020) suggests that high input activity (e.g., task-evoked activity) saturates neuronal response, resulting in reduction of signal variability and inter-regional connectivity. In contrast, for low input activity (e.g., resting-state activity) neuronal responses remain at lower levels, while signal variability and inter-regional connectivity increase. In the context of adaptive processing, presenting novel (i.e., non-adaptation condition) versus familiar (i.e., adaptation condition) stimuli relates to higher versus lower neuronal input, respectively. Lower neuronal input for adaptation may relate to repetition suppression (i.e., decreased fMRI responses) in visual cortex and increased occipito-parietal connectivity, reflecting an alertness state in which the brain decreases its responses to familiar stimuli but remains ready to respond to novel information.

Further, the predictive coding theory provides a framework for integrating our results on repetition suppression and enhancement (De Gardelle et al. 2013; Segaert et al. 2013). According to this theory, higher-order brain regions generate a prediction for incoming sensory information that is compared against the sensory input in early sensory areas. The difference between the predicted and the sensory input constitutes the prediction error and is used to revise the predictions (Friston 2005; Huang and Rao 2011; Shipp 2016). In the context of adaptive processing, repeated stimuli are associated with smaller prediction errors than novel stimuli, resulting in higher responses in sensory areas for novel versus familiar stimuli, consistent with repetition suppression in visual cortex (Auksztulewicz and Friston 2016; Grotheer and Kovács 2016).

Our multimodal imaging approach sheds light into the functional interactions between sensory-adaptation and perceptual-memory networks that support adaptive processing and behavior. Our experimental design and data analyses pipelines allowed us to control for potential confounds. First, it is unlikely that differences in fMRI adaptation and functional connectivity between conditions (adaptation vs. non-adaptation) could be due to differences in attention or stimulus expectation, as participants engaged with a demanding RSVP task at fixation (Larsson et al. 2016) with similar performance across conditions. Second, we denoised the fMRI signals from contributions of motion, veins, and multiband acquisition using a state-of-the-art ICA denoising procedure (Griffanti et al. 2014). Third, we controlled for task-induced inflation in connectivity and a potential neurovascular component in BOLD differences using deconvolution methods (Cole et al. 2019). Future work would be valuable in addressing the following potential limitations. Our results provide correlational evidence for the role of sensory-adaptation and perceptual-memory networks in adaptive processing. Future work employing interventional approaches (e.g., transcranial direct current stimulation) that have been shown to enhance learning (Sczesny-Kaiser et al. 2016; Frangou et al. 2018) and brain plasticity (Stagg et al. 2011, 2014; Meinzer et al. 2012) could interrogate causal relationships between brain networks and behavior. Further, our MRS investigations involved measurements of GABA during rest rather than task performance. Future work could employ functional MRS approaches to measure GABA during stimulus presentation and interrogate the dynamics of GABAergic inhibition that underlie adaptive behavior.

In sum, our findings provide evidence for functional brain network interactions that support adaptive processing and behavior: a sensory-adaptation network (including V1, IPS, dlPFC) that is involved in adaptive processing in response to stimulus repetition, and a perceptual-memory network (including PMN, dmPFC) that relates to adaptive behavior as measured by the tilt aftereffect. Further, our results suggest that GABAergic inhibition may gate interactions between these networks to support adaptive processing and behavior in the human brain, providing new insights into the role of memory systems in efficient sensory processing and adaptive behavior.

## Data Availability

The data underlying this article are available in [Apollo], at https://doi.org/10.17863/CAM.70738.

## Funding

Biotechnology and Biological Sciences Research Council (grant numbers H012508, BB/P021255/1 to Z.K.); Wellcome Trust (grant number 205067/Z/16/Z to Z.K.); European Community’s Seventh Framework Programme (FP7/2007-2013 under agreement PITN-GA-2011-290011 to Z.K.). For the purpose of open access, the author has applied for a CC BY public copyright license to any author accepted manuscript version arising from this submission.

## Notes

We would like to thank Uzay Emir for advice on MRS data analyses, and the MR physics and radiographer teams at the Wolfson Brain Imaging Unit and the Cognition and Brain Sciences Unit, for their support and help with data collection. *Conflict of Interest:* All authors declare that they have no conflict of interest.

## Supplementary Material

Karlaftis_SI_final_bhab160
