# Supplementary material for: Functional Interactions between Sensory and Memory Networks for Adaptive Behavior
Source: Cereb Cortex. 2021 Jun 29;31(12):5319–30. doi: 10.1093/cercor/bhab160 (PMC8568003; doi:10.1093/cercor/bhab160)
Supplement: Karlaftis_SI_final_bhab160 [file karlaftis_si_final_bhab160.docx]

# **Supplementary Figures**


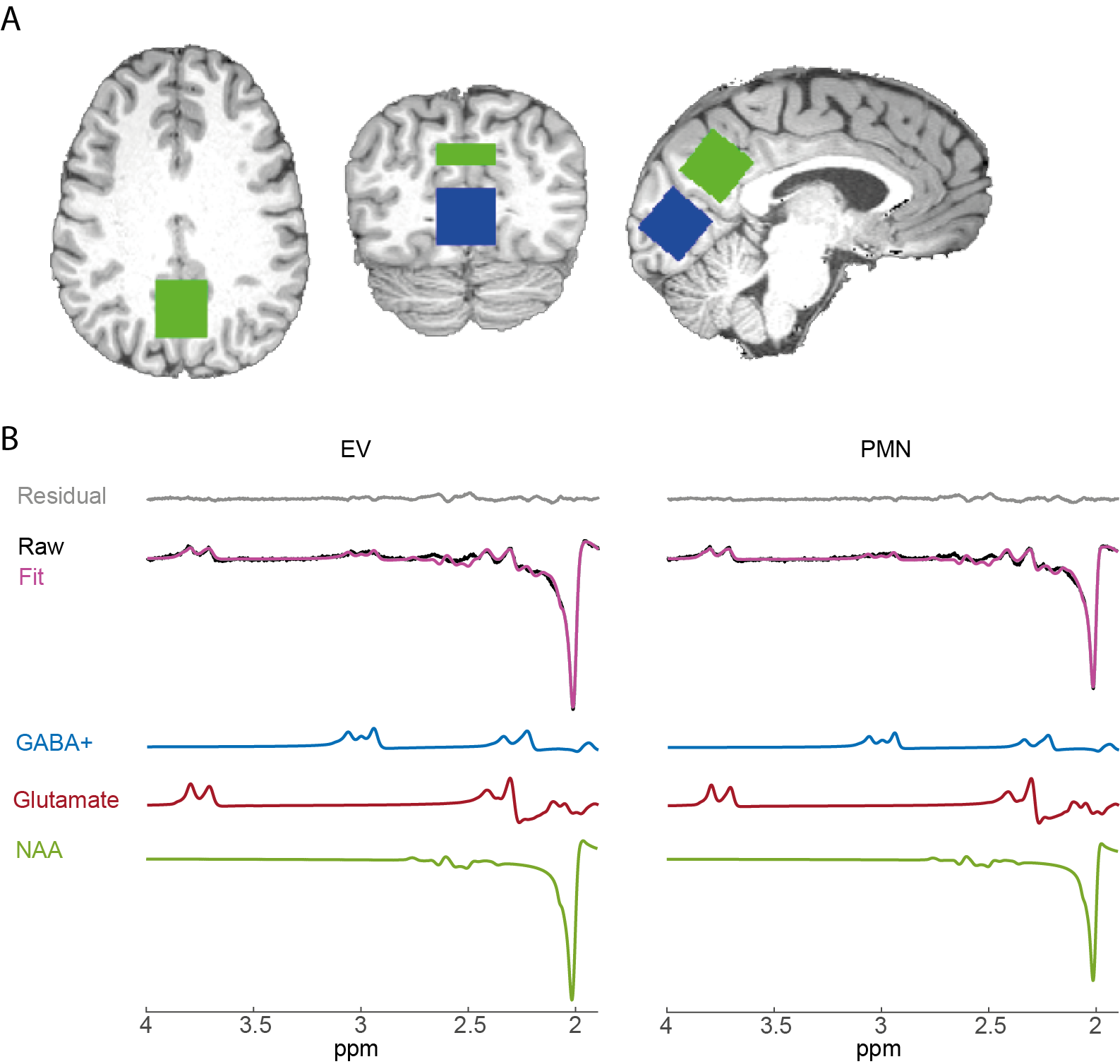


**Figure S1. MRS voxels.** (A) Placement: We positioned the MRS voxels manually using anatomical landmarks on the acquired T1 scan. We illustrate the placements of the EV (blue) and the PMN (green) MRS voxels in the native space of a representative participant. The EV voxel was placed medially in the occipital lobe with the lower face aligned with the cerebellar tentorium and as posterior as possible towards the occipital pole given the voxel dimensions. The PCC voxel was placed in the medial parietal lobe and rotated in the sagittal plane to align with a line connecting the genu and splenium of the corpus callosum. (B) MRS spectra: Spectra from the EV and PMN voxels for a representative participant. We show the LC model fit, the residual and the respective fits for GABA+, Glutamate and NAA.


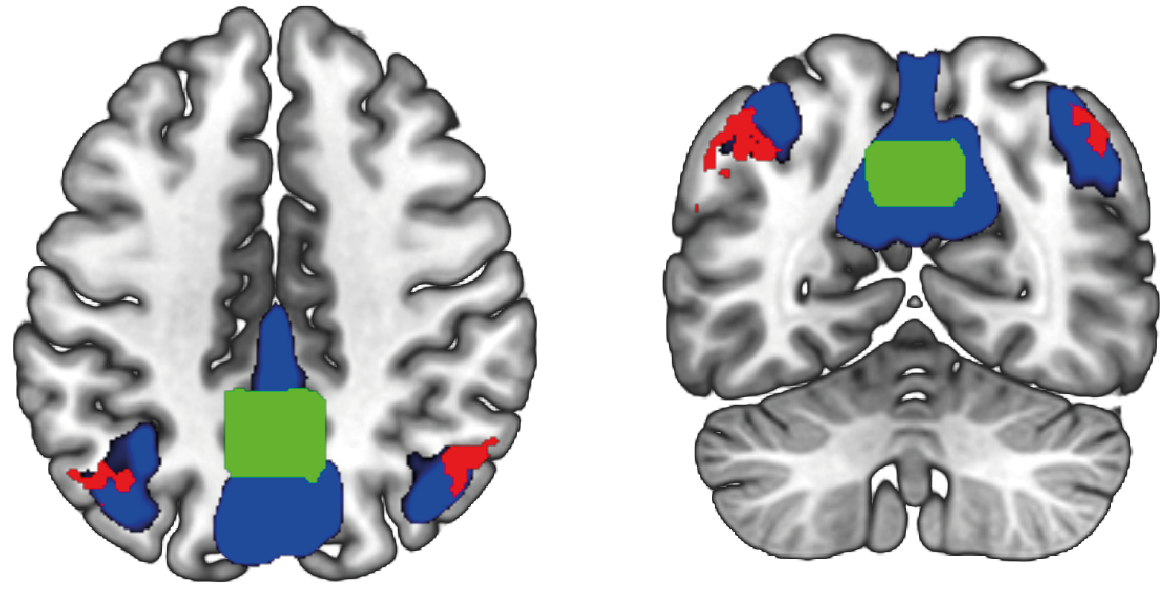


**Figure S2. PMN localisation**: Spatial overlap between the PMN as identified in the ICA (blue; thresholded at z=1.7), the angular clusters as identified in the GLM analysis with behaviour (red; Table S4) and the PMN MRS voxel in MNI space (green). The data are displayed in neurological convention (left is left) in axial (z=44) and coronal (y=-63) views.


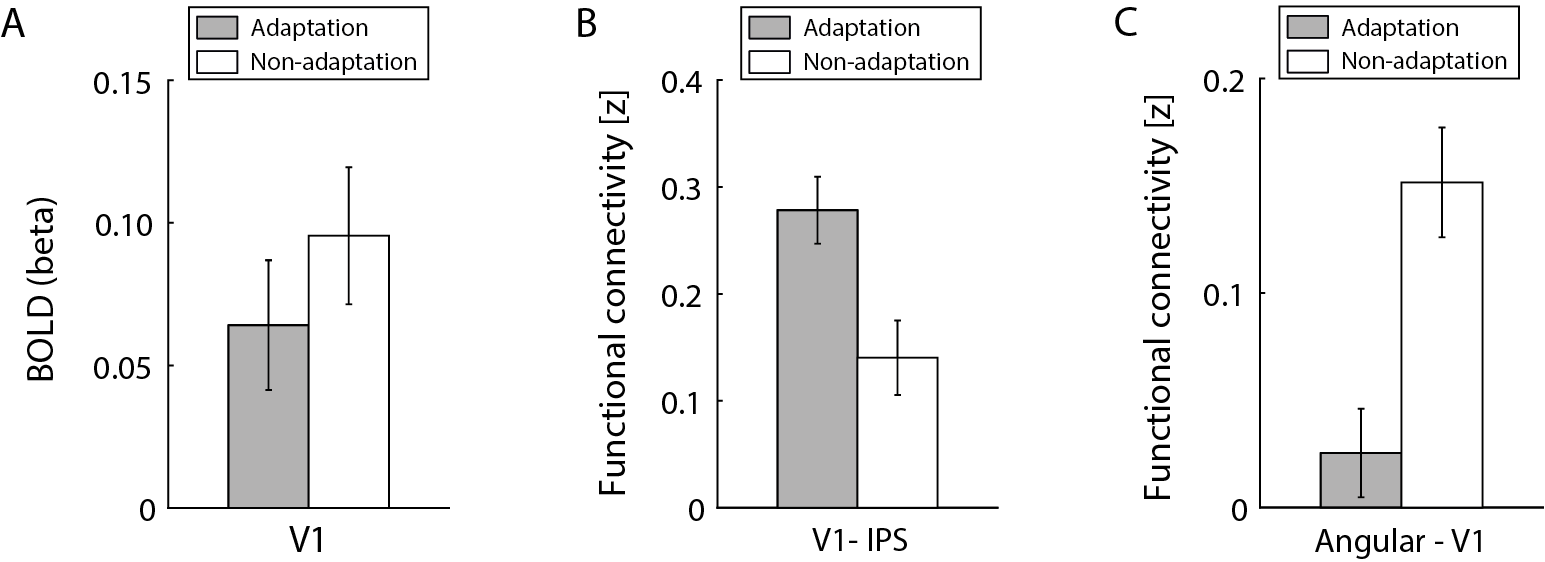


**Figure S3. Replication in an independent group**: (A) BOLD analysis: lower BOLD (beta) in V1 for adaptation than non-adaptation. (B) Seed-based functional connectivity from V1: higher V1 – IPS connectivity for adaptation than non-adaptation (Table S7). (C) Seed-based functional connectivity from angular gyrus: lower Angular – V1 connectivity for adaptation than non-adaptation (Table S8). Error bars indicate standard error of the mean across participants.

# **Supplementary Tables**

**Table S1.** **MRS** **quality measures**: Absolute Cramer-Rao lower bound (CRLB), linewidth and signal-to-noise ratio (SNR) are shown for the two MRS voxels.

| MRS voxel | MRS quality measure | Mean | Std |
| --- | --- | --- | --- |
| EV | CRLB | 9.40 | 0.99 |
|  | Linewidth | 7.17 | 0.81 |
|  | SNR | 47.02 | 4.31 |
| PMN | CRLB | 9.85 | 0.82 |
|  | Linewidth | 6.32 | 0.35 |
|  | SNR | 42.69 | 4.78 |

**Table S2.** **Control analyses for correlations with MRS GABA+/NAA**: (a) Whole brain GLM with EV GABA+, (b) whole brain GLM with PMN GABA+, (c) seed-based functional connectivity from V1 with EV GABA+, and (d) seed-based functional connectivity from angular gyrus with PMN GABA+. Pearson correlations (r, p) for each significant cluster when: using α-corrected GABA+ referenced to water, controlling for tissue composition within the MRS mask by regressing out the CSF percentage, controlling for Glu levels in the MRS voxel, and controlling for MRS data quality (CRLB, linewidth, SNR).

| Analysis | Cluster | Control |  | r | p |
| --- | --- | --- | --- | --- | --- |
| *Whole brain GLM*  *with EV GABA+* | **IFJ** | GABA+/water |  | 0.45 | 0.024 |
|  |  | %CSF |  | 0.69 | <0.001 |
|  |  | Glu |  | 0.70 | <0.001 |
|  |  | CRLB |  | 0.67 | <0.001 |
|  |  | Linewidth |  | 0.71 | <0.001 |
|  |  | SNR |  | 0.67 | <0.001 |
|  | **IFS** | GABA+/water |  | 0.47 | 0.017 |
|  |  | %CSF |  | 0.72 | <0.001 |
|  |  | Glu |  | 0.78 | <0.001 |
|  |  | CRLB |  | 0.66 | <0.001 |
|  |  | Linewidth |  | 0.74 | <0.001 |
|  |  | SNR |  | 0.73 | <0.001 |
| *Whole brain GLM*  *with PMN GABA+* | **MFG** | GABA+/water |  | 0.71 | <0.001 |
|  |  | %CSF |  | 0.84 | <0.001 |
|  |  | Glu |  | 0.71 | <0.001 |
|  |  | CRLB |  | 0.82 | <0.001 |
|  |  | Linewidth |  | 0.83 | <0.001 |
|  |  | SNR |  | 0.81 | <0.001 |
|  | **IPS** | GABA+/water |  | 0.79 | <0.001 |
|  |  | %CSF |  | 0.82 | <0.001 |
|  |  | Glu |  | 0.66 | <0.001 |
|  |  | CRLB |  | 0.82 | <0.001 |
|  |  | Linewidth |  | 0.85 | <0.001 |
|  |  | SNR |  | 0.80 | <0.001 |
| *Seed-based connectivity from V1 with EV GABA+* | **Postcentral, Precentral** | GABA+/water |  | -0.41 | 0.042 |
|  |  | %CSF |  | -0.58 | 0.002 |
|  |  | Glu |  | -0.52 | 0.007 |
|  |  | CRLB |  | -0.52 | 0.007 |
|  |  | Linewidth |  | -0.57 | 0.003 |
|  |  | SNR |  | -0.53 | 0.007 |
|  | **Postcentral** | GABA+/water |  | -0.45 | 0.026 |
|  |  | %CSF |  | -0.57 | 0.003 |
|  |  | Glu |  | -0.56 | 0.004 |
|  |  | CRLB |  | -0.56 | 0.004 |
|  |  | Linewidth |  | -0.59 | 0.002 |
|  |  | SNR |  | -0.54 | 0.006 |
| *Seed-based connectivity from angular gyrus with PMN GABA+* | **IPS, V3a, V3b** | GABA+/water |  | 0.50 | 0.016 |
|  |  | %CSF |  | 0.43 | 0.041 |
|  |  | Glu |  | 0.56 | 0.005 |
|  |  | CRLB |  | 0.45 | 0.031 |
|  |  | Linewidth |  | 0.46 | 0.026 |
|  |  | SNR |  | 0.44 | 0.037 |

**Table S3.** **Whole-brain GLM (adaptation vs. non-adaptation)**: Significance was determined using cluster-correction at p=0.05 FWER after an initial cluster-extent threshold at an uncorrected p=0.005. Clusters were labelled using the Probabilistic map of Visual Topography (Wang, Mruczek, Arcaro, & Kastner, 2015) or the Automated Anatomical Labeling (AAL) and Human Connectome Project (HCP) atlases (Glasser et al., 2016; Tzourio-Mazoyer et al., 2002) for clusters beyond the visual cortex. The number of voxels, the MNI coordinates, the p-value and the t-value of the peak voxel are shown for each cluster.

| Cluster location | Hem. | number of voxels | Peak voxel | | | | |  |
| --- | --- | --- | --- | --- | --- | --- | --- | --- |
|  |  |  | **x** | **y** | **z** | **p_FWER-corr_** | **t-value** | |
| V1v, V1d, V2v | L | 99 | -12 | -86 | -12 | <0.001 | -4.92 | |
| V3d | L | 51 | -18 | -94 | 18 | 0.003 | -4.83 | |
| V1d, V2d, V3d, V3a | R | 102 | 18 | -92 | 14 | <0.001 | -4.74 | |
| V3v, VO2 | L | 35 | -18 | -70 | -10 | 0.040 | -4.43 | |
| MFG (dorsal area 9/46) | R | 41 | 22 | 38 | 34 | 0.014 | -4.24 | |

**Table S4.** **Whole-brain GLM with perceptual adaptation index as regressor**: Significance was determined using cluster-correction at p=0.05 FWER after an initial cluster-extent threshold at an uncorrected p=0.005. Clusters were labelled using the Probabilistic map of Visual Topography (Wang et al., 2015) or the AAL and HCP atlases (Glasser et al., 2016; Tzourio-Mazoyer et al., 2002) for clusters beyond the visual cortex. The number of voxels, the MNI coordinates, the p-value and the t-value of the peak voxel are shown for each cluster.

| Cluster location | Hem. | number of voxels | Peak voxel | | | | |  |
| --- | --- | --- | --- | --- | --- | --- | --- | --- |
|  |  |  | **x** | **y** | **z** | **p_FWER-corr_** | **t-value** | |
| Cerebellum | L | 78 | -36 | -68 | -36 | <0.001 | -5.49 | |
| Angular | L | 204 | -44 | -64 | 48 | <0.001 | -5.41 | |
| Angular | R | 168 | 46 | -62 | 50 | <0.001 | -5.05 | |
| SFG (anterior area 9) | L | 65 | -16 | 60 | 22 | <0.001 | -4.31 | |
| MTG | R | 44 | 60 | -26 | 2 | 0.007 | -3.93 | |

**Table S5.** **Seed-based functional connectivity from V1**: Significance was determined using cluster-correction at p=0.05 FWER after an initial cluster-extent threshold at an uncorrected p=0.005. Clusters were labelled using the Probabilistic map of Visual Topography (Wang et al., 2015) or the AAL and HCP atlases (Glasser et al., 2016; Tzourio-Mazoyer et al., 2002) for clusters beyond the visual cortex. The number of voxels, the MNI coordinates, the p-value and the t-value of the peak voxel are shown for each cluster.

| Cluster location | Hem. | number of voxels | Peak voxel | | | | |  |
| --- | --- | --- | --- | --- | --- | --- | --- | --- |
|  |  |  | **x** | **y** | **z** | **p_FWER-corr_** | **t-value** | |
| IPS | R | 179 | 22 | -70 | 32 | <0.001 | 5.23 | |
| IPS | L | 67 | -18 | -62 | 48 | 0.035 | 4.31 | |

**Table S6.** **Seed-based functional connectivity from angular gyrus**: Significance was determined using cluster-correction at p=0.05 FWER after an initial cluster-extent threshold at an uncorrected p=0.005. Clusters were labelled using the Probabilistic map of Visual Topography (Wang et al., 2015) or the AAL and HCP atlases (Glasser et al., 2016; Tzourio-Mazoyer et al., 2002) for clusters beyond the visual cortex. The number of voxels, the MNI coordinates, the p-value and the t-value of the peak voxel are shown for each cluster.

| Cluster location | Hem. | number of voxels | Peak voxel | | | | |  |
| --- | --- | --- | --- | --- | --- | --- | --- | --- |
|  |  |  | **x** | **y** | **z** | **p_FWER-corr_** | **t-value** | |
| Cerebellum | R | 79 | 42 | -62 | -26 | 0.012 | -6.92 | |
| STG | L | 127 | -62 | -36 | 20 | <0.001 | -5.47 | |
| Cerebellum | L, R | 71 | 12 | -50 | -26 | 0.025 | -5.00 | |
| V1v, V2v, VO | R | 137 | 24 | -62 | -4 | <0.001 | -4.86 | |
| IPS | L | 165 | -14 | -80 | 36 | <0.001 | -4.54 | |
| Fusiform, Cerebellum | L | 72 | -28 | -44 | -18 | 0.023 | -4.47 | |
| V3v, VO, PHP | L | 137 | -26 | -58 | -8 | <0.001 | -4.43 | |
| STG | R | 111 | 68 | -28 | 22 | 0.001 | -4.36 | |

**Table S7.** **Replication in an independent group: Seed-based functional connectivity from V1**: Significance was determined using cluster-correction at p=0.05 FWER after an initial cluster-extent threshold at an uncorrected p=0.025. Clusters were labelled using the Probabilistic map of Visual Topography (Wang et al., 2015) or the AAL and HCP atlases (Glasser et al., 2016; Tzourio-Mazoyer et al., 2002) for clusters beyond the visual cortex. The number of voxels, the MNI coordinates, the p-value and the t-value of the peak voxel are shown for each cluster.

| Cluster location | Hem. | number of voxels | Peak voxel | | | | |  |
| --- | --- | --- | --- | --- | --- | --- | --- | --- |
|  |  |  | **x** | **y** | **z** | **p_FWER-corr_** | **t-value** | |
| IPL | L | 452 | -39.6 | -48.8 | 50 | 0.002 | 5.75 | |
| IPS | L | 785 | -6.8 | -75.2 | 50 | <0.001 | 5.44 | |
| V3a | R | 360 | 13.2 | -85.6 | 34.8 | 0.012 | 5.20 | |
| LO, V3d | L | 767 | -36.4 | -85.6 | 10 | <0.001 | 4.93 | |
| Precuneus, Cuneus | L, R | 390 | 5.2 | -71.2 | 26.8 | 0.006 | 4.69 | |
| IPL, Supramarginal | L | 519 | -56.4 | -32 | 46.8 | <0.001 | 4.55 | |

**Table S8.** **Replication in an independent group: Seed-based functional connectivity from angular** **gyrus:** Significance was determined using cluster-correction at p=0.05 FWER after an initial cluster-extent threshold at an uncorrected p=0.025. Clusters were labelled using the Probabilistic map of Visual Topography (Wang et al., 2015) or the AAL and HCP atlases (Glasser et al., 2016; Tzourio-Mazoyer et al., 2002) for clusters beyond the visual cortex. The number of voxels, the MNI coordinates, the p-value and the t-value of the peak voxel are shown for each cluster.

| Cluster location | Hem. | number of voxels | Peak voxel | | | | |  |
| --- | --- | --- | --- | --- | --- | --- | --- | --- |
|  |  |  | **x** | **y** | **z** | **p_FWER-corr_** | **t-value** | |
| V1v, V1d | R | 420 | 11.6 | -78.4 | 1.2 | 0.003 | -5.39 | |
| Angular | R | 299 | 41.2 | -69.6 | 46.8 | 0.048 | -5.07 | |

**Table S9.** **Whole-brain GLM with EV GABA+** **as regressor**: Significance was determined using cluster-correction at p=0.05 FWER after an initial cluster-extent threshold at an uncorrected p=0.005. Clusters were labelled using the Probabilistic map of Visual Topography (Wang et al., 2015) or the AAL and HCP atlases (Glasser et al., 2016; Tzourio-Mazoyer et al., 2002) for clusters beyond the visual cortex. The number of voxels, the MNI coordinates, the p-value and the t-value of the peak voxel are shown for each cluster.

| Cluster location | Hem. | number of voxels | Peak voxel | | | | |  |
| --- | --- | --- | --- | --- | --- | --- | --- | --- |
|  |  |  | **x** | **y** | **z** | **p_FWER-corr_** | **t-value** | |
| IFJ | L | 41 | -50 | 10 | 38 | 0.014 | 4.43 | |
| IFS (posterior) | L | 39 | -48 | 16 | 22 | 0.020 | 4.30 | |

**Table S10.** **Whole-brain GLM with PMN GABA+** **as regressor**: Significance was determined using cluster-correction at p=0.05 FWER after an initial cluster-extent threshold at an uncorrected p=0.005. Clusters were labelled using the Probabilistic map of Visual Topography (Wang et al., 2015) or the AAL and HCP atlases (Glasser et al., 2016; Tzourio-Mazoyer et al., 2002) for clusters beyond the visual cortex. The number of voxels, the MNI coordinates, the p-value and the t-value of the peak voxel are shown for each cluster.

| Cluster location | Hem. | number of voxels | Peak voxel | | | | |  |
| --- | --- | --- | --- | --- | --- | --- | --- | --- |
|  |  |  | **x** | **y** | **z** | **p_FWER-corr_** | **t-value** | |
| MFG (dorsal area 9/46) | R | 35 | 34 | 42 | 42 | 0.035 | 4.08 | |
| IPS | R | 69 | 36 | -72 | 38 | <0.001 | 3.86 | |

**Table S11.** **Seed-based functional connectivity from V1 with EV GABA+** **as regressor**: Significance was determined using cluster-correction at p=0.05 FWER after an initial cluster-extent threshold at an uncorrected p=0.005. Clusters were labelled using the Probabilistic map of Visual Topography (Wang et al., 2015) or the AAL and HCP atlases (Glasser et al., 2016; Tzourio-Mazoyer et al., 2002) for clusters beyond the visual cortex. The number of voxels, the MNI coordinates, the p-value and the t-value of the peak voxel are shown for each cluster.

| Cluster location | Hem. | number of voxels | Peak voxel | | | | |  |
| --- | --- | --- | --- | --- | --- | --- | --- | --- |
|  |  |  | **x** | **y** | **z** | **p_FWER-corr_** | **t-value** | |
| Postcentral, Precentral | R | 161 | 34 | -28 | 66 | <0.001 | -4.73 | |
| Postcentral | R | 80 | 12 | -50 | 72 | 0.010 | -4.56 | |

**Table S12.** **Seed-based functional connectivity from angular** **gyrus with PMN GABA+** **as regressor**: Significance was determined using cluster-correction at p=0.05 FWER after an initial cluster-extent threshold at an uncorrected p=0.005. Clusters were labelled using the Probabilistic map of Visual Topography (Wang et al., 2015) or the AAL and HCP atlases (Glasser et al., 2016; Tzourio-Mazoyer et al., 2002) for clusters beyond the visual cortex. The number of voxels, the MNI coordinates, the p-value and the t-value of the peak voxel are shown for each cluster.

| Cluster location | Hem. | number of voxels | Peak voxel | | | | |  |
| --- | --- | --- | --- | --- | --- | --- | --- | --- |
|  |  |  | **x** | **y** | **z** | **p_FWER-corr_** | **t-value** | |
| IPS, V3a, V3b | R | 336 | 10 | -84 | 28 | <0.001 | 5.13 | |
| SFG (medial area 8B), Cingulate | L, R | 70 | 6 | 26 | 42 | 0.024 | -4.54 | |

**Table S13.** **Seed-based functional connectivity from EV MRS voxel with EV GABA+** **as regressor**: Significance was determined using cluster-correction at p=0.05 FWER after an initial cluster-extent threshold at an uncorrected p=0.005. Clusters were labelled using the Probabilistic map of Visual Topography (Wang et al., 2015) or the AAL and HCP atlases (Glasser et al., 2016; Tzourio-Mazoyer et al., 2002) for clusters beyond the visual cortex. The number of voxels, the MNI coordinates, the p-value and the t-value of the peak voxel are shown for each cluster.

| Cluster location | Hem. | number of voxels | Peak voxel | | | | |  |
| --- | --- | --- | --- | --- | --- | --- | --- | --- |
|  |  |  | **x** | **y** | **z** | **p_FWER-corr_** | **t-value** | |
| Cingulate, SMA | L, R | 161 | 10 | -22 | 46 | <0.001 | -5.66 | |
| Postcentral | R | 383 | 26 | -46 | 76 | <0.001 | -5.56 | |
| Paracentral, Precentral | L | 139 | -26 | -20 | 78 | <0.001 | -5.11 | |
| Precuneus | L | 108 | -14 | -44 | 70 | 0.001 | -4.94 | |
| Precentral, Postcentral | R | 127 | 44 | -24 | 68 | <0.001 | -4.43 | |
| Cingulate, Precuneus | R | 64 | 8 | -40 | 56 | 0.047 | -4.23 | |

**Table S14.** **Seed-based functional connectivity from PMN MRS voxel with PMN GABA+** **as regressor**: Significance was determined using cluster-correction at p=0.05 FWER after an initial cluster-extent threshold at an uncorrected p=0.005. Clusters were labelled using the Probabilistic map of Visual Topography (Wang et al., 2015) or the AAL and HCP atlases (Glasser et al., 2016; Tzourio-Mazoyer et al., 2002) for clusters beyond the visual cortex. The number of voxels, the MNI coordinates, the p-value and the t-value of the peak voxel are shown for each cluster.

| Cluster location | Hem. | number of voxels | Peak voxel | | | | |  |
| --- | --- | --- | --- | --- | --- | --- | --- | --- |
|  |  |  | **x** | **y** | **z** | **p_FWER-corr_** | **t-value** | |
| IPS, V3a | R | 130 | 30 | -82 | 24 | <0.001 | 5.25 | |
